# Effects of Neutrophil Extracellular Traps on Bovine Mammary Epithelial Cells *in vitro*

**DOI:** 10.3389/fimmu.2019.01003

**Published:** 2019-05-17

**Authors:** Zhengkai Wei, Jingjing Wang, Yanan Wang, Chaoqun Wang, Xiao Liu, Zhen Han, Yunhe Fu, Zhengtao Yang

**Affiliations:** ^1^College of Life Sciences and Engineering, Foshan University, Foshan, China; ^2^College of Veterinary Medicine, Jilin University, Changchun, China

**Keywords:** NETs, bovine mastitis, BMECs, pyroptosis, NLRP3

## Abstract

Bovine mastitis is a common infectious disease which causes huge economic losses in dairy cattle. Bovine mammary epithelial cell (BMEC) damage usually directly causes the decrease of milk production, which is one of the most important causes of economic loss. NETs, novel effector mechanisms, are reported to exacerbate the pathogenesis of several inflammatory diseases. NETs formation has also been observed in the milk and mammary glands of sheep. However, the effects and detailed mechanisms of NETs on BMEC damage remain unclear. Thus, we aim to examine the effects of NETs on BMECs *in vitro*, and further to investigate the detail mechanism. In this study, the cytotoxicity of NETs on BMECs was determined using lactic dehydrogenase (LDH) levels in culture supernatants. Histone-induced BMEC damage was examined by flow cytometry and immunofluorescence analysis. The activities of caspase 1, caspase 3, caspase 11, and NLRP3 was detected using western blotting and immunohistochemical analysis. The results showed that NETs and their component histone significantly increased cytotoxicity to BMECs, suggesting the critical role of NETs, and their component histone in BMEC damage. In addition, histone could also induce necrosis, pyroptosis, and apoptosis of BMECs, and the mechanisms by which histone leads to BMEC damage occurred via activating caspase 1, caspase 3, and NLRP3. Altogether, NETs formation regulates inflammation and BMEC damage in mastitis. Inhibiting excess NETs formation may be useful to ameliorate mammary gland damage associated with mastitis.

## Introduction

Mastitis is one of the most prevalent and costly diseases in dairy herds. It causes enormous economic losses due to reduced milk production and quality, and treatment costs (1, 2). A large number of studies have shown that many microorganisms can induce the occurrence of mastitis (3, 4). It is the result of attempts of the host to eliminate invading microorganisms. The innate immune system is known to be the first line of defense against microorganisms and plays a critical role in the initiation of an inflammatory response in the mammary gland (5). Neutrophils are most abundant leukocyte cells in the innate immune system of the host (6). They have the ability to eliminate invading pathogens through phagocytosis and death (7). In recent years, a new killing mechanism has been discovered in neutrophils: neutrophil extracellular traps (NETs) (8). NETs are mainly composed of a DNA framework and attachment proteins including histone, elastase, and myeloperoxidase (MPO). NETs formation can be triggered by many kinds of stimuli, such as bacteria, virus, fungus, and parasites. The release of NETs plays a curial role in pathogen control and elimination (8). In recent studies, the formation of NETs has been observed in milk and in the mammary alveoli of mastitic sheep and cows (9, 10), but the effects of NETs on mammary epithelial cells has not being reported.

Recent studies have shown that NETs are a double-edged sword of the innate immune system (11, 12). In other words, when NETs play a curial role in infection, excessive NETs release or hindered NETs degradation can also lead to tissue damage. It is reported that NETs exacerbated liver injury during liver ischemia/reperfusion, and inhibiting NETs formation can attenuate liver ischemia/reperfusion injuries (12). NETs and their associated histone could lead to the damage of epithelial and endothelial cells (13). Normally, histone provides stability to the chromatin within the nucleus, but they can be released once the disease occurs, such as sepsis or autoimmune disease (14). Released histone works as damage-associated molecular pattern proteins, activating a series of host immune responses and resulting in endothelial and epithelial cytotoxicity (14). NETs, particularly histone, could lead to human, alveolar, and epithelial cell injury and lung tissue destruction (13). Furthermore, NETs and their component histone have also been reported to be related to tubular necrosis and organ injury in ischemic AKI (15). However, the potential role of NETs in mammary gland damage remains unclear. Thus, this study aims to investigate the effects of NETs and their components on BMEC damage, and further to clarify the potential mechanisms underlying mammary epithelial cells damage.

## Materials and Methods

### Materials

Caspase-1 antibody (AB1872) and caspase 3 antibody (AB4051, Abcam) were purchased from Abcam Bio Co., Ltd. Caspase-11 antibody (NB120-10454, Novus) was purchased from Novus Biologicals. NLRP3 antibody was purchased from Boster Bio Co., Ltd, China. Histone type II A and PMA were purchased from the Sigma-Aldrich. DNase I and FLUOS-Annexin V/PI kits were acquired from Hoffmann-La Roche.

### Isolation, Culture, and Identification of BMECs

BMECs were isolated from alveolar tissues of lactating cows by a procedure previously described (16, 17). In brief, three healthy lactating Chinese Holstein cows (3–4 years old) were first examined to identify milk, somatic cell counts, and clinical mastitis. Then, healthy cows were sacrificed, and mammary glands were collected within 2 h. The tissue was cut into 1-mm^3^ pieces and washed with D-Hank's solution until there was no milk remaining. The tissue pieces were laid flat on culture flasks and subjected to inverted cultivation at 37°C with 5% CO_2_ for 4 h. Then, the culture flasks were reversed and cultured at 37°C with 5% CO_2_ following the addition of 5 mL of DMEM/F12 media containing 10% FBS (HyClone), 100 U/mL of penicillin, streptomycin (100 mg/mL), and 1 mg/mL of amphotericin B (Invitrogen, Carlsbad, CA, USA). The media were replaced once every 48 h until the cells had spread fully across the culture flask. Rapid trypsinization with trypsin (0.25, 01% EDTA-2Na) and differential adhesion were used to obtain the BMECs.

To identify the purity of the BMECs, fluorescence confocal analysis was carried out. The BMECs were cultured briefly on cover glasses (pre-treated with poly-_L_-lysine, 0.1 mg/mL, Sigma-Aldrich) and fixed with 4 % (w/v) paraformaldehyde for 20 min. The cells were then permeabilized with 0.1% Triton X-100 for 15 min and blocked in 3% goat serum/PBS, followed by incubation with antibody to cytokeratin-18 (Rabbit anti-cytokeratin 18, Boster Bio Co., Ltd, China) and a second antibody of goat anti-rabbit IgG-FITC conjugated (5 μg/mL in PBS, Bioworld Technology Inc.). The DNA was stained with DAPI. Finally, the samples were observed with scanning confocal microscopy (Olympus FluoView FV1000), and the image was processed by using FV10-ASW 4.1. All animal studies were approved by the Animal Welfare and Research Ethics Committee at Jilin University (JLU20170330).

### Cytotoxicity Assay

Lactate dehydrogenase (LDH) released into the supernatant was determined using an LDH Cytotoxicity Assay kit® (Beyotime Biotechnology, China). In brief, BMECs (2 × 10^6^) were incubated with NET (484 ng/mL), histone (50–200 μg/mL) or NET (484 ng/mL) treated with DNase I (1.5 mg/mL) for 16 h. Lactate dehydrogenase (LDH) released into the supernatant by the BMECs was examined according to the manufacturer's protocols. The cytotoxicity of BMECs was calculated as follows: cytotoxicity (%) = (exp. value-low control)/(high control-low control) × 100. BMECs viability was determined by the Cell Counting Kit-8 and Microplate reader at a wavelength of 450 nm. In brief, BMECs (2 × 10^6^) were incubated with NET (342 ng/mL), NET (342 ng/mL) + DNase I (1.5 mg/mL), NET (342 ng/mL) + DNase I (3 mg/mL) or NET (342 ng/mL) or histone (50–200 μg/mL) for 16 h. The viability of BMECs was calculated as follows: Cell viability (%) = (exp. Value–control)/(exp. Control without stimulus- control) × 100. For bovine neutrophil isolation, three adult healthy cows were bled from the caudal vein, and the blood was collected in tubes containing heparin. Neutrophils were isolated using a commercially available Bovine PMN isolation kit® (TianJin HaoYang Biological Manufacture CO., China). To perform induction and isolation of the NETs, 2×10^6^ neutrophils were co-cultured with 50 nM of PMA for 4 h, and the medium was removed. Then, 2 mL of RPMI was added to the well plate and collected. After centrifugation at 100× g for 5 min, NETs in the supernatant were collected and quantified using a Picogreen ds DNA kit (Invitrogen), as previously described (13). For digestion of the NETs, newly isolated NETs were pretreated with DNase I (Roch, Germany) for 20 min at 37°C.

### Flow Cytometry and Fluorescence Confocal Microscopy Analyses

BMECs were incubated with histone (50–200 μg/mL) for 16 h. Then, the BMECs were stained with FLUOS-Annexin V/PI according to the manufacturer's protocols (Roche) and examined using flow cytometry (BD FACSCalibur, USA). The data was analyzed by BD CellQuest Pro Software. BMECs without staining with FLUOS-Annexin V/PI were used as unstained controls. BMECs without histone-treatment were used as controls. For fluorescence confocal microscopy analysis, BMECs were seeded on 6-well plate, and treated with histone (50–200 μg/mL) for 16 h. After incubation, BMECs was treated with trypsin-EDTA solution (Hyclone), stained with FLUOS-Annexin V/PI and observed with fluorescence confocal microscopy. Finally, the image was processed using FV10-ASW 4.1.

### Western Blot Analysis

Total proteins from the histone-stimulated BMECs were extracted using mammalian, protein extraction reagent (Thermo). Protein concentration was determined using a BCA protein assay kit (Thermo). Prepared samples containing proteins (30 μg) were separated by 15% SDS-PAGE and transferred onto a PVDF membrane (85 Volt, 45 min). The membranes were blocked with 3% BSA at room temperature for 2 h and then probed with primary antibodies at 4°C overnight. Subsequently, the membranes were washed three times with TBST and probed with specific secondary antibodies solution for 2 h at room temperature. Finally, the membranes were washed three more times, and blots were developed using the ECL plus Western Blotting Detection System (GE Healthcare, Chalfont St Giles, UK). The blots were analyzed by Image J Software. The following primary antibodies were used: caspase 1 (1 μg/mL in TBST, AB1872, Abcam), caspase 3 (1 μg/mL in TBST, AB4051, Abcam), caspase 11 (1 μg/mL in TBST, NB120-10454, Novus), and NLRP3 (0.2 μg/mL in TBST, Boster, China).

### Immunocytochemistry Analysis

BMECs were cultured briefly on cover glasses (pre-treated with poly-L-lysine, 0.1 mg/mL, Sigma-Aldrich) and incubated with histone (50–200 μg/mL) for 16 h. The cells were fixed with 4% (w/v) paraformaldehyde for 30 min and washed three times. After that, the samples treated with antigen retrieval, non-specifically blocked with 3% H_2_O_2_ in methanol for 40 min, 5% goat serum/PBS blocked for 45 min and followed by incubation with antibody to caspase 1 (2.5 μg/mL, AB1872, Abcam), caspase 3 (2.5 μg/mL, AB4051, Abcam), caspase 11 (2.5 μg/mL, NB120-10454, Novus), and NLRP3 (0.5 μg/mL, Boster, China) overnight at 4°C and incubated with the secondary antibody (Maxim, KIT9710, China) at room temperature for 30 min. Finally, the samples were visualized with DAB (Maxim, DAB-0031, China) and counterstained with hematoxylin.

### Statistical Analysis

The data were analyzed using GraphPad 5.0 (GraphPad InStat Software, USA). Comparisons among the groups were performed using a one-way analysis of variance (ANOVA) with Tukey's multiple comparison tests. Western blotting was analyzed using ImageJ software (National Institutes of Health, USA). Data were expressed as the mean ±S.E.M. *P*-values of < 0.05 were considered significant (^*^P < 0.05).

## Results

### Isolation and Culture of BMECs

**Figure 1:**
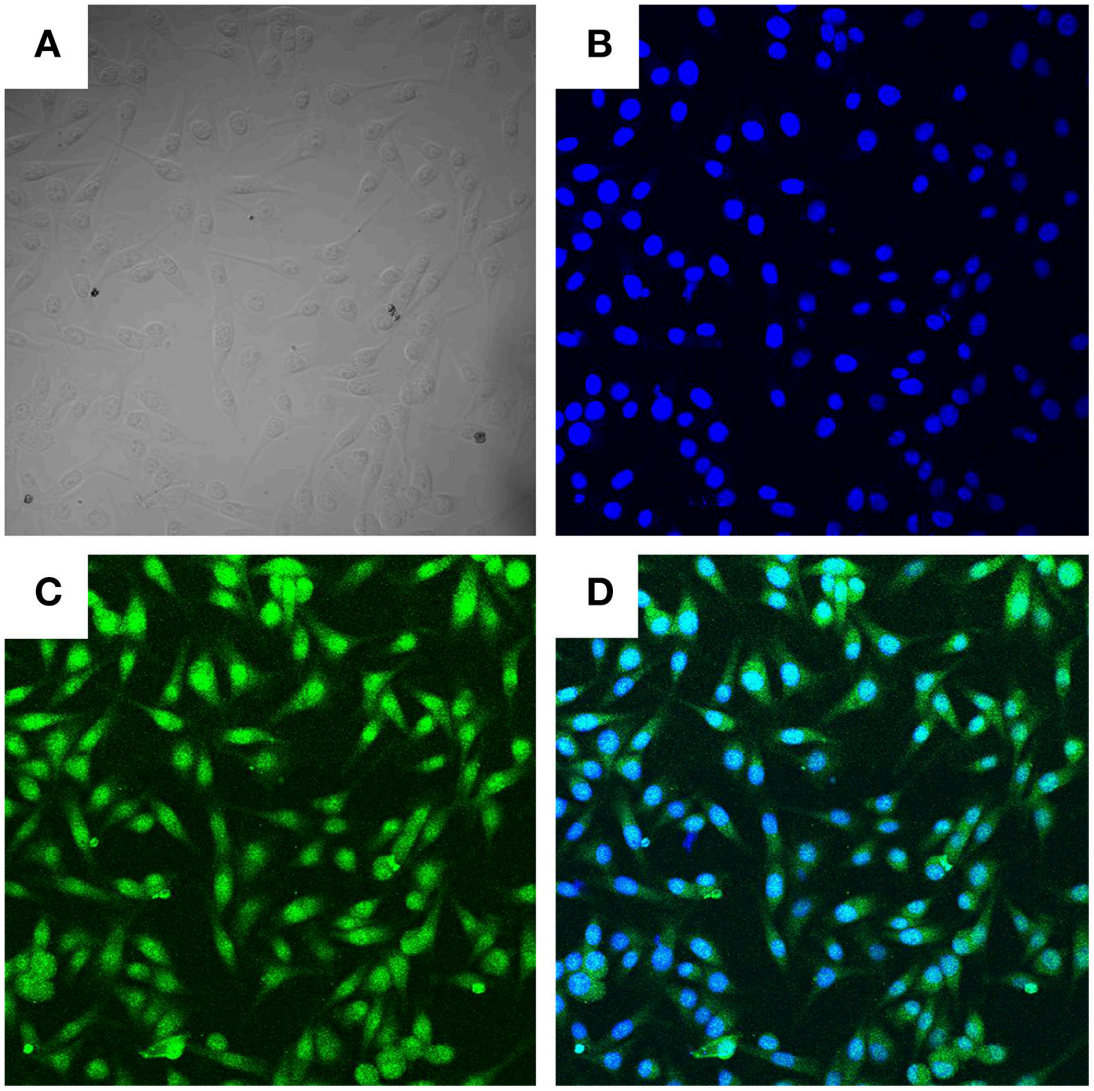
Morphologic observation of BMECs (400×). Three independent experiments were carried out by fluorescence confocal analyses. **(A)** Light microscope observation of BMECs. **(B)**. DAPI staining (Blue, DNA). **(C)** Cytokeratin−18 (Green). **(D)** Merge of **(B,C)**.

Trypsinization of the bovine mammary gland was used to isolate BMECs. The purity of BMECs was authenticated by fluorescence confocal microscopy. As shown in Figure 1, we observed BMECs morphology (A), DNA staining (B), and cytokeratin-18 expression (C). All of these results showed that we successfully isolated BMECs, which were the foundation for the subsequent research.

### NETs Induced Cytotoxicity in BMECs

**Figure 2:**
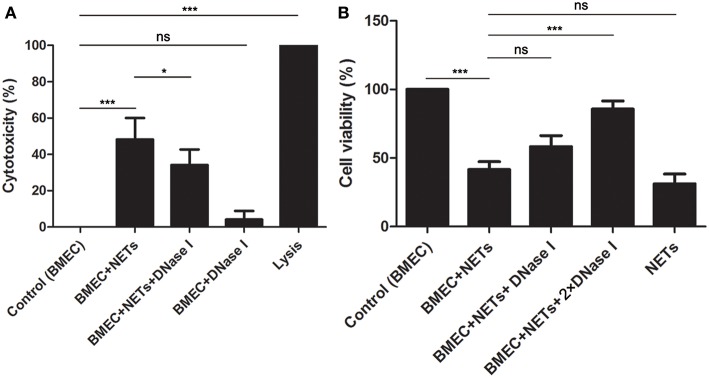
NETs induced cytotoxicity in BMECs. **(A)**The cytotoxicity of BMECs was determined after treatment with NET (484 ng/mL), NET (484 ng/mL) + DNase I or DNase I for 16 h. Lysis provided by LDH Cytotoxicity Assay Kits was used as positive controls. **(B)** The viability of BMECs was detected by Cell Counting Kit-8. Briefly, BMECs were treated with NET (342 ng/mL), NET (342 ng/mL) + DNase I (1.5 mg/mL), NET (342 ng/mL) + DNase I (3 mg/mL), or NET (342 ng/mL) for 16 h. After washed for three times, the samples were treated with 10 μL CCK-8, incubated for 2 h and examined microplate reader at wavelength at 450 nm. Date are presented as mean ± SEM (*n* = 5). *P* < 0.05 were considered significant (^*^P < 0.05, ^***^P < 0.001, and “ns” means not significant).

To investigate the effects of NETs on BMECs, BMECs were incubated with NETs, NETs treated with DNase I or DNase I for 16 h. It was found that NET (484 ng/mL) stimulation increased LDH release, but NETs treated with DNase I significantly decrease the cytotoxicity of BMECs (Figure 2A, *n* = 5). Moreover, the CCK-8 assays also showed that NET (342 ng/mL) induced BMEC damage but were markedly alleviated by DNase I (Figure 2B, *n* = 5). These results showed that NETs significantly increased the cytotoxicity of the BMECs, suggesting the critical role of NETs in BMEC damage.

### Histone Increased the Cytotoxicity of BMECs

**Figure 3:**
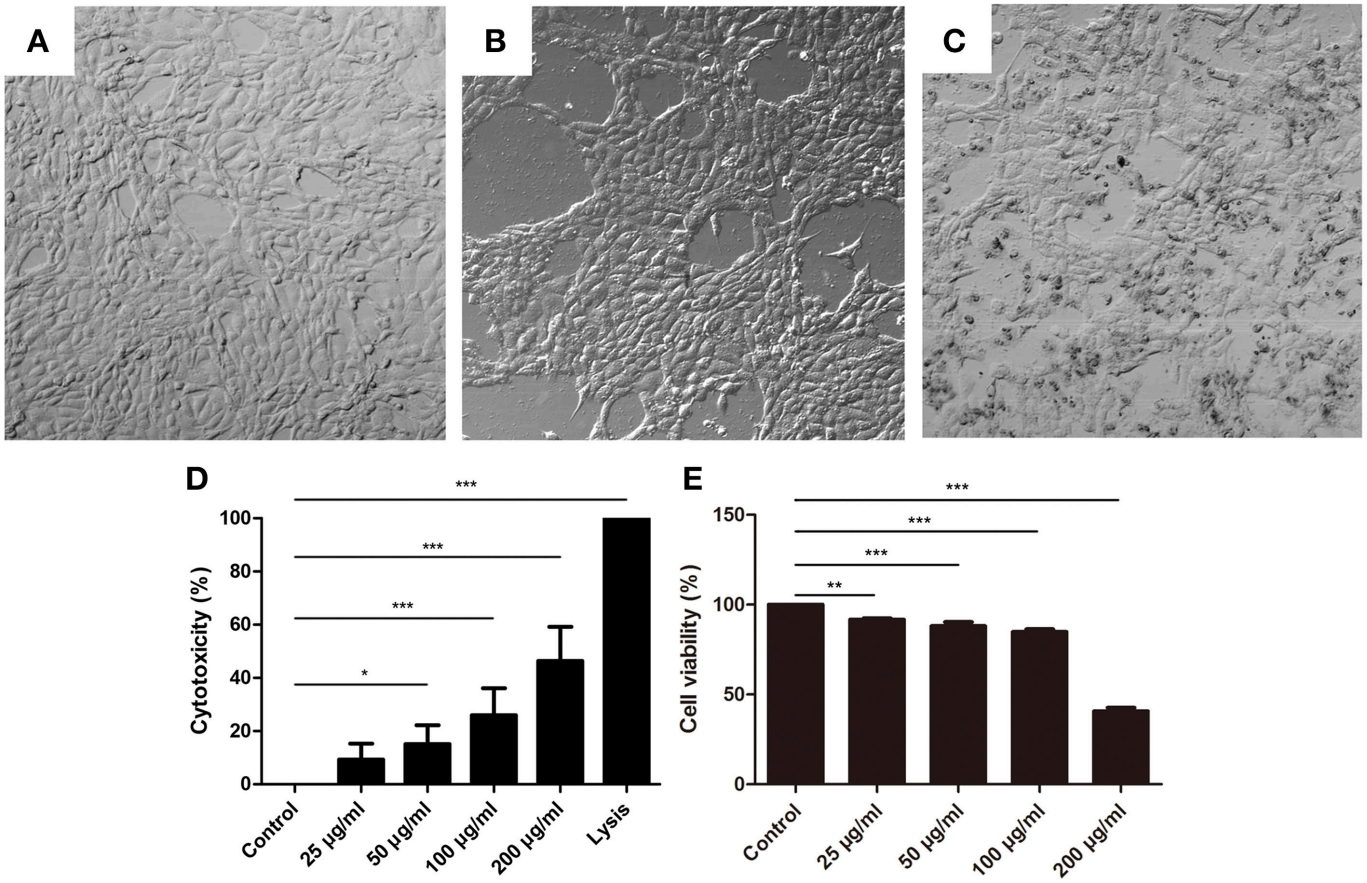
Histone caused BMECs death. **(A–C)** The changes of BMECs morphology were measured after treatment with different concentrations of histone for 16 h (400×). Three independent experiments were carried out by light microscope analyses. **(A)** Controls. **(B)** BMECs treated with histone (100 μg/mL). **(C)** BMECs treated with histone (200 μg/mL). **(D)** Histone induced cytotoxicity to BMECs. The cytotoxicity of BMECs was determined by LDH Cytotoxicity Assay Kits after treatment with different concentrations for 16 h. Lysis provided by LDH Cytotoxicity Assay Kits was used as positive controls. **(E)** Effects of histone on the viability of BMECs. BMECs were incubated with histone and examined by CCK-8 kits. Date are presented as mean ± SEM (*n* = 5). *P* < 0.05 were considered significant (^*^P < 0.05, ^**^P < 0.01, and ^***^P < 0.001).

To further investigate the effects of NET components on BMECs, BMECs were incubated with histone for 16 h. The results showed that the morphology of BMECs was significantly changed by histone treatment (Figures 3B,C, *n* = 5) compared to control groups (Figure 3A). In addition, the cytotoxicity of BMECs was determined after treatment with different concentrations for 16 h. It was found that histone also significantly increased the cytotoxicity of the BMECs in a dose-dependent manner (Figures 3D,E, *n* = 5), which suggested the important role of histone-induced BMEC damage.

### Histone Induced BMECs Pyroptosis

**Figure 4:**
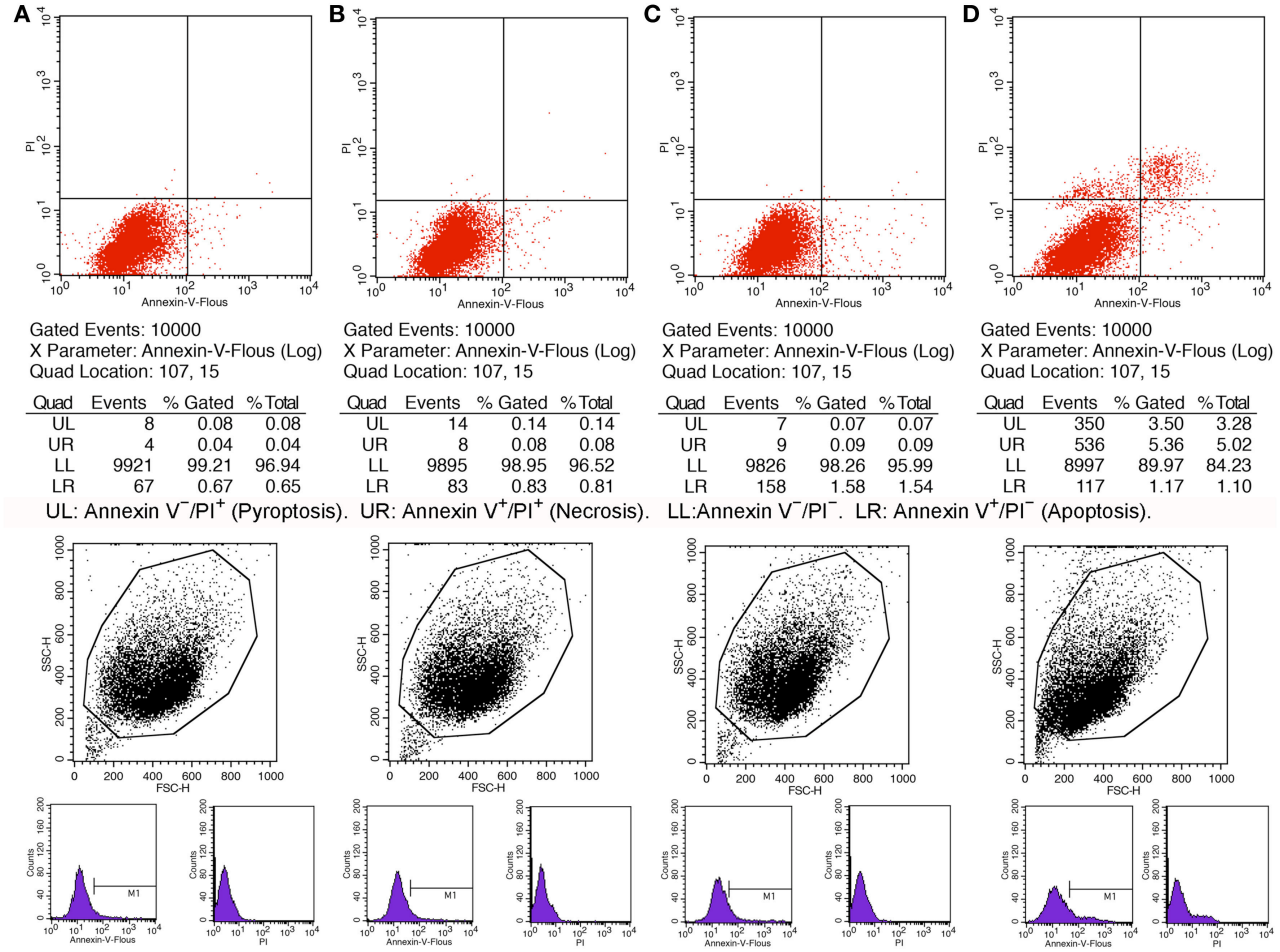
Flow cytometry analyses of histone caused BMECs apoptosis, necrosis, and pyroptosis. BMECs were incubated with stimulated histone for 16 h. Then, the BMECs were stained FLUOS-Annexin V/PI according to the manufacturer's protocols and were examined by Flow cytometry. The results represent that Annexin V^−^/PI^−^ (LL) and Annexin V^−^/PI^+^ (Pyroptosis, UL), Annexin V^+^/PI^−^ (Apoptosis, LR), and Annexin V^+^/PI^+^ (Necrosis, UR). **(A)** Controls. **(B)** BMECs treated with histone (50 μg/mL). **(C)**. BMECs treated with histone (100 μg/mL). **(D)** BMECs treated with histone (200 μg/mL). Histone caused BMECs necrosis (5.02%), pyroptosis (3.28%), and apoptosis (1.10%) in a dose-dependent. Three independent experiments were carried out by flow cytometry analyses.

**Figure 5:**
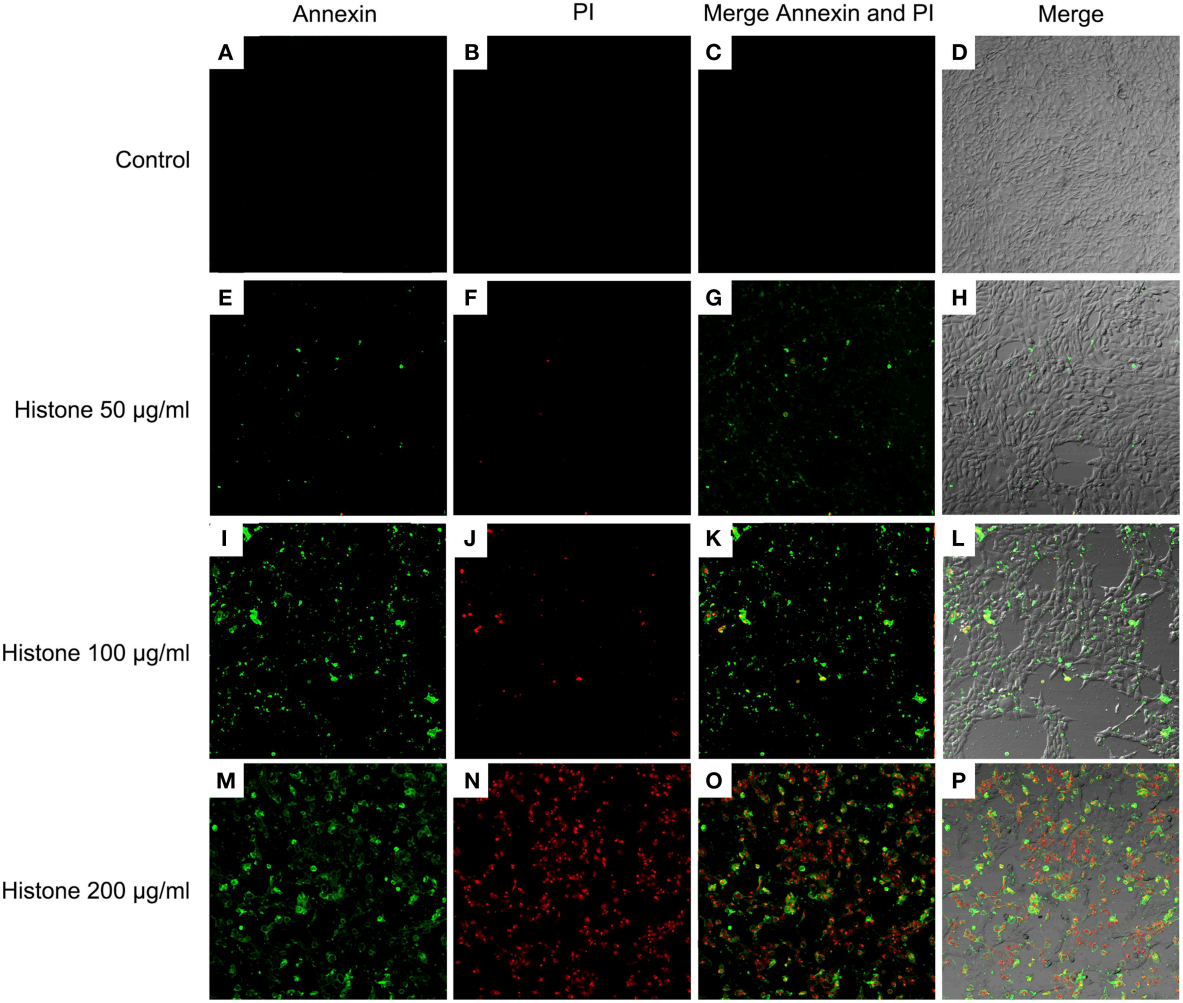
Fluorescence confocal microscopy analyses of histone caused BMECs apoptosis, necrosis, and pyroptosis (400×). BMECs were incubated with stimulated histone for 16 h. Then, the BMECs were stained FLUOS-Annexin V/PI according to the manufacturer's protocols and were examined by fluorescence confocal microscopy analyses. Three independent experiments were carried out by fluorescence confocal analyses.

**Figure 6:**
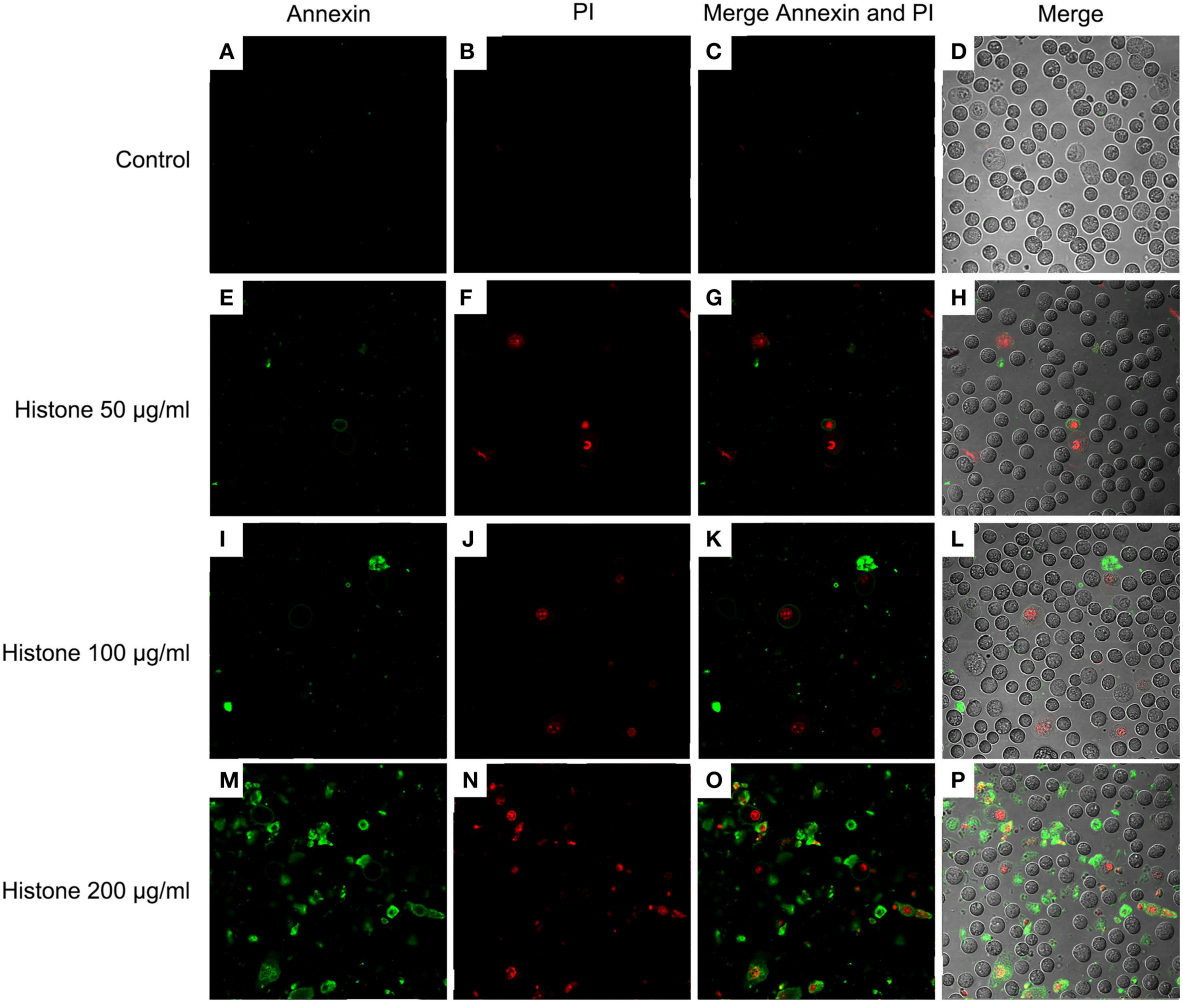
Fluorescence confocal microscopy analyses of histone caused BMECs apoptosis, necrosis, and pyroptosis (400×). BMECs were incubated with stimulated histone for 16 h. Then, the BMECs were dissociated and stained FLUOS-Annexin V/PI according to the manufacturer's protocols and were examined by fluorescence confocal microscopy analyses. Three independent experiments were carried out by fluorescence confocal analyses.

Knowing the toxic effects of histone on BMECs, we next investigated how histone induced BMEC damage. Flow cytometry analysis has been used as a vital tool to identify cell death types, such as Annexin V^−^/PI^+^ (Pyroptosis), Annexin V^+^/PI^−^ (Apoptosis), and Annexin V^+^/PI^+^ (Necrosis) (18). It has been shown that histone (200 μg/mL) treatment caused BMEC necrosis (UR; 5.02%), pyroptosis (UL; 3.28%), and apoptosis (LR; 1.10 %). Specifically, pyroptosis was first observed in the BMEC damage process (Figure 4, *n* = 3). Furthermore, the results of the flow cytometry analysis showed that histone-induced BMECs necrosis, pyroptosis, and apoptosis was also confirmed by fluorescence confocal microscopy analyses (Figures 5, 6, *n* = 3). The results showed that histone significantly induced pyroptosis, apoptosis, and necrosis.

### Histone-Induced BMECs Pyroptosis Is Dependent on Activation of Caspase 1, Caspase 3, and NLRP3

**Figure 7:**
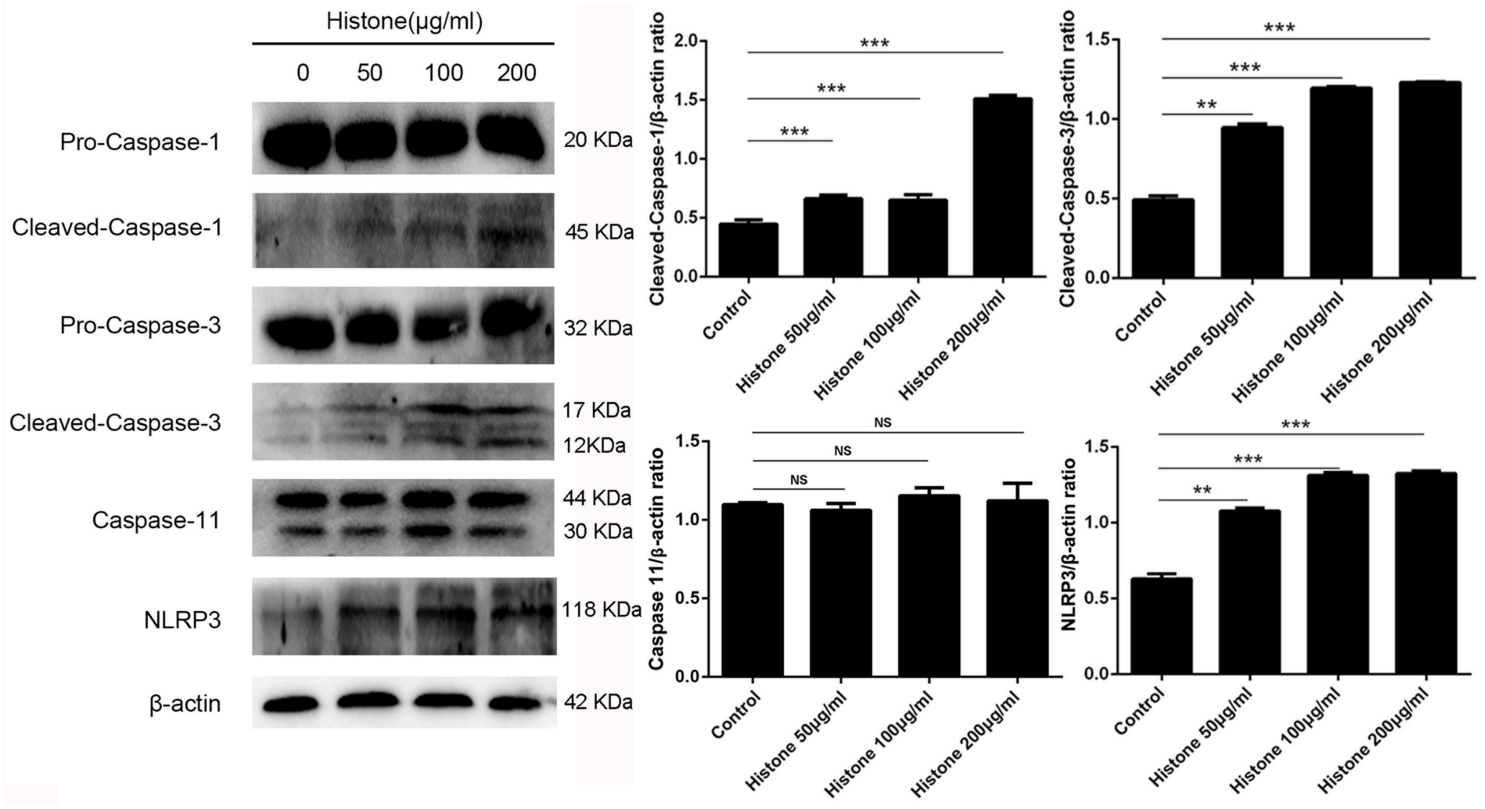
Histone induce BMECs pyroptosis is dependent on activations of caspase 1, caspase 3 and NLRP3. BMECs were incubated with stimulated histone for 16 h, and the activities of these proteins were determined by western blotting. Date are presented as mean ± SEM (*n* = 5). *P*-values of < 0.05 were considered significant (^**^P < 0.01, ^***^P < 0.001, and “ns” means not significant).

**Figure 8:**
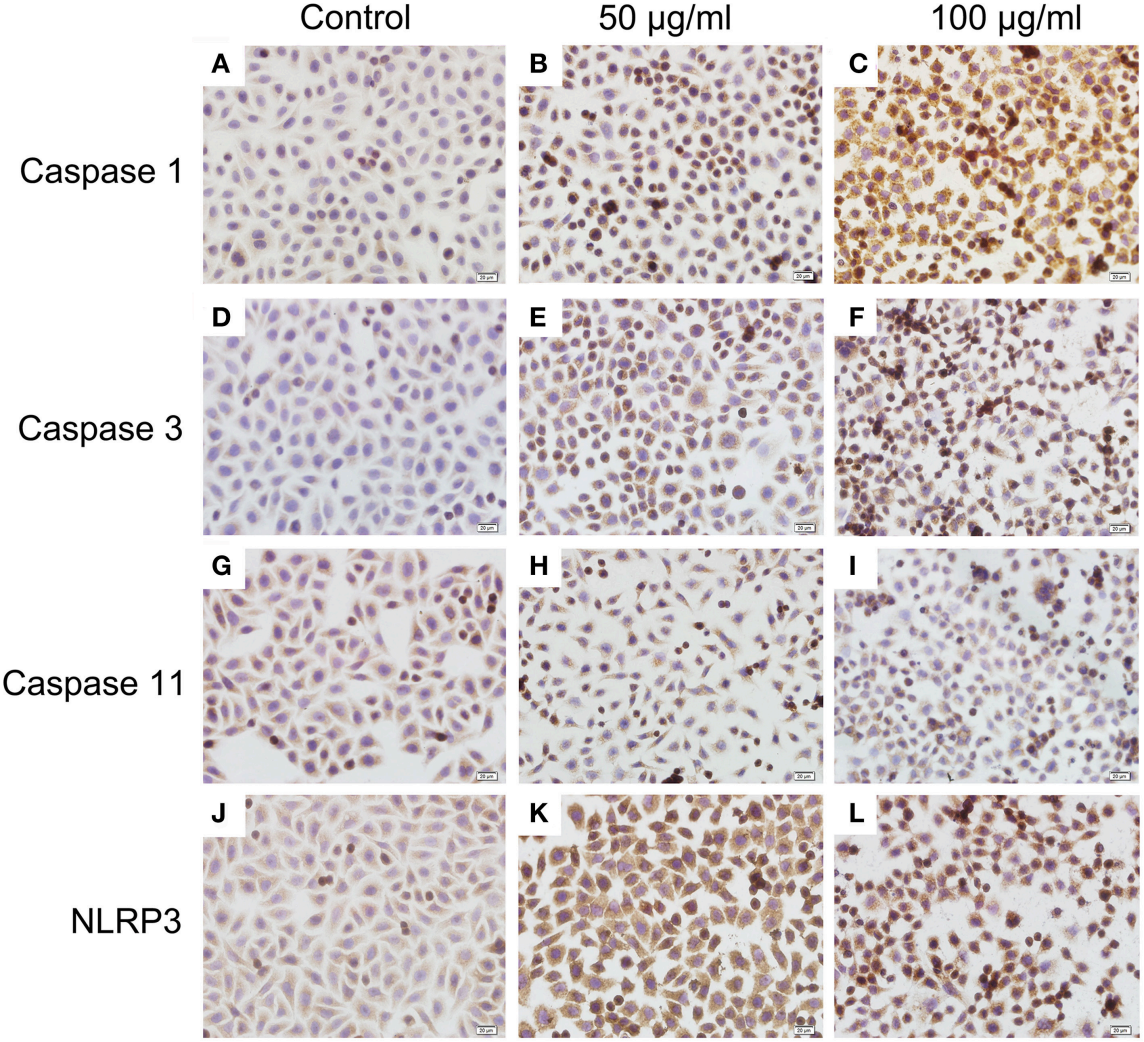
Immunocytochemistry analysis of histone induced activations of caspase 1, caspase 3, and NLRP3 (400×). BMECs were cultured briefly on cover glasses (pre-treated with poly-L –lysine, 0.1 mg/mL, Sigma-Aldrich) and incubated with histone (50 and 100 μg/mL) for 16 h. The samples were visualized with DAB, counterstained with hematoxylin and observed by inverted microscope. Three independent experiments were carried out by light microscope analyses.

To investigate the mechanism of histone-induced BMEC pyroptosis, we examined the key molecules involved in the pyroptosis process. Using western blotting, we found that histone markedly increased the activation of caspase 1, caspase 3, and NLRP3 but did not change the activation of caspase 11 (Figure 7, *n* = 5). To further confirm the results of western blotting, immunocytochemistry analysis was carried out to investigate these molecules in histone-induced BMEC pyroptosis. The results showed that histone significantly activated caspase 1, caspase 3, and NLRP3 but did not significantly change the activation of caspase 11 (Figure 8, *n* = 3). All these results suggest that histone-induced BMEC pyroptosis is dependent on the activation of caspase 1, caspase 3, and NLRP3.

## Discussion

Neutrophils are one of most abundant and effective leukocyte populations protecting the mammary gland (19, 20). NETs, an effector mechanisms of neutrophils, have been observed in the milk and mammary glands of sheep (9) as well as milk of *Staphylococcus aureus*-mastitis in dairy cows (21). It is also showed that NETs formation could not be inhibited by milk (22). These data suggest that there is no doubt about the release of NETs in the milk and the mammary gland environment. However, excess NETs formation is also reported to exacerbate the pathogenesis of many inflammatory diseases (23, 24). In mastitis, the effects of NETs on bovine mammary epithelial cells have not been previously investigated. In the present study, we found that NETs significantly increased the cytotoxicity of BMECs, and DNase I markedly decreased NETs-induced cytotoxicity, suggesting that DNA plays an import role in NETs-induced BMEC cytotoxicity. However, NETs-induced BMEC cytotoxicity could not be completely inhibited by DNase I, which means the role of other NET protein components are involved in this process. Next, we examined the effects of other components of NETs on BMECs. It was found that histone also increased the LDH release of BMECs in a dose-dependent manner, which is in accordance with the effects of NETs and histone on A549 mouse lung epithelial cells (13). The above results reveal that NETs and their component histone also play a key role in the BMEC damage associated with mastitis.

Knowing that histone is the most abundant protein within NETs and their toxic effects on BMECs, we next aimed to investigate how histone induced BMEC damage. Multiple reports have shown that apoptosis and necrosis are the typical cell death types resulting in BMEC damage (25, 26). In this study, histone not only caused necrosis and apoptosis of BMECs but also caused pyroptosis, especially pyroptosis first observed in the BMECs. Pyroptosis is a caspase-1-dependent or independent programmed inflammatory cell death, which is a critical component of several inflammatory diseases (27, 28). Inflammasomes, such as NLRP3, also play an important role in the caspase-1-dependent pyroptosis process (28). Furthermore, several caspase signaling pathways are related to cell pyroptosis, necrosis, and apoptosis (28–30). To further investigate the mechanism of histone-induced BMEC damage, we then examined the key molecules involved in the process of pyroptosis, necrosis, and apoptosis. Indeed, we found that histone significantly increased the activation of caspase 1, caspase 3, and NLRP3 in BMECs. It is interesting to note that histone did not change the activation of caspase 11, suggesting that histone-induced BMEC pyroptosis is a caspase-1-dependent process. All these results further confirmed our findings that pyroptosis, necrosis and apoptosis are involved in histone-induced BMEC damage associated with mastitis. Although histone-induced pyroptosis, necrosis and apoptosis partially explain the cytotoxicity associated with BMEC damage, whether this mechanism is involved in bovine mammary gland damage *in vivo* remains unknown.

In summary, NET formation was firstly reported to be involved in mammary epithelial cell damage *in vitro*. It was also found that histone, a component of NETs, plays a key role in BMEC damage, and the mechanisms by which histone-induced pyroptosis, necrosis, and apoptosis of BMECs activate via caspase 1, caspase 3, and NLRP3. All these results suggest that inhibiting excess NET formation may be useful to ameliorate mammary gland damage associated with mastitis.

## Ethics Statement

All animal studies were approved by the Animal Welfare and Research Ethics Committee at Jilin University.

## Author Contributions

ZY, YF, and ZW designed the project and experiments. ZW, JW, CW, XL, and ZH carried out most of the experiments. YF and ZW wrote the manuscript. ZW, YW, and ZH carried out the statistical analysis and prepared figures. YF and ZY co-corresponded this paper. All authors reviewed the manuscript.

### Conflict of Interest Statement

The authors declare that the research was conducted in the absence of any commercial or financial relationships that could be construed as a potential conflict of interest.
